# First systematic review of the last 30 years of research on sweetpotato: elucidating the frontiers and hotspots

**DOI:** 10.3389/fpls.2024.1428975

**Published:** 2024-07-04

**Authors:** Xiaoqing Meng, Tingting Dong, Zongyun Li, Mingku Zhu

**Affiliations:** The Key Laboratory of Biotechnology for Medicinal Plant of Jiangsu Province, School of Life Sciences, Jiangsu Normal University, Xuzhou, Jiangsu, China

**Keywords:** bibliometrics, CiteSpace, research trend, research hotspot, sweetpotato, visualization analysis

## Abstract

Sweetpotato is an economically important crop, and it has various advantages over other crops in addressing global food security and climate change. Although substantial articles have been published on the research of various aspects of sweetpotato biology, there are no specific reports to systematically crystallize the research achievements. The current review takes the lead in conducting a keyword-centric spatiotemporal dimensional bibliometric analysis of articles on sweetpotato research using CiteSpace software to comprehensively clarify the development status, research hotspot, and development trend in the past 30 years (1993–2022). Quantitative analysis was carried out on the publishing countries, institutions, disciplines, and scholars to understand the basic status of sweetpotato research; then, visual analysis was conducted on high-frequency keywords, burst keywords, and keyword clustering; the evolution of major research hotspots and the development trend in different periods were summarized. Finally, the three main development stages—preliminary stage (1993–2005), rapid stage (2006–2013), and diversified mature stage (2014–2022)—were reviewed and analyzed in detail. Particularly, the development needs of sweetpotato production in improving breeding efficiency, enhancing stress tolerance, coordinating high yield with high quality and high resistance, and promoting demand were discussed, which will help to comprehensively understand the development dynamics of sweetpotato research from different aspects of biological exploration.

## Introduction

Declining agricultural land, increasing global population, and uncertain adverse environmental changes pose complex and daunting challenges to global food production. Although much progress has been made in crop yield improvement, most of the existing efforts have focused on aboveground traits (mainly cereal crops), which limits the further improvement of the yield potential, but exploring the root systems is expected to overcome the bottleneck. Consequently, it is imperative to stabilize and enhance crop yield by exploring the underground traits of root and tuber crops, which will enable a truly inclusive green revolution (Khan et al., 2016).

Root and tuber crops contain large amounts of carbohydrates and different levels of proteins and vitamins, making them an indispensable part of the human diet. Particularly, potato (tuber crops), sweetpotato, and cassava (tuberous root crops) are among the most important food crops globally, as well as core cereals and legumes. The storage organs of potato and sweetpotato are similar, but there are significant differences in botany: the former is formed from tubers, while the latter is developed from fibrous roots into expanded tuberous roots (also known as storage roots). The genus *Ipomoea* has the most species in the Convolvulaceae family, with approximately 800 species (Wood et al., 2020). These species are widely used in agriculture, industry, medicine, and ornamental plants, as well as evolutionary and molecular genetic studies (Meira et al., 2012; Yan et al., 2022). For instance, *Ipomoea aquatica* not only is one of the most abundant sources of carotenoids and chlorophylls but also contains a sufficient supply of the most essential amino acids and excellent bioelements, which can be comparable to conventional foodstuffs, and *Ipomoea nil* has a wide range of applications in garden purposes and genetic studies due to diverse flower colors and pigmentation patterns (Meira et al., 2012). Particularly, sweetpotato [*Ipomoea batatas* (L.) Lam.] is the only crop plant with starch storage roots among approximately 1,650 major tropical species in Convolvulaceae (Meira et al., 2012; Arisha et al., 2020).

Sweetpotato, a dicotyledonous hexaploid plant species (2n = 6x = 90, B1B1B2B2B2B2), has long been the seventh largest food crop worldwide among all food crops based on production, mainly produced by Asia and Africa, and China has consistently been the biggest sweetpotato producer (Liu, 2017; Yang et al., 2017). It offers many unique advantages such as strong stress resistance, tolerance to barrenness, high and stable yield, rich nutritional components, and diversified applications (Liu, 2017; Alam, 2021). Sweetpotato is cultivated in more than 120 countries and regions from the temperate zone south of 40°N to the tropics (Luo et al., 2023). It is primarily planted in arid, salinized, hilly regions with poor marginal conditions (Liu, 2017; Bach et al., 2021), which is of great significance for stabilizing and improving agricultural production. In terms of nutrition, sweetpotato is an excellent source of starches, proteins, vitamins, dietary fibers, flavonoids, carotenoids, minerals, and many other antioxidant compounds (Johnson and Pace, 2010; Alam, 2021). Additionally, sweetpotato leaves (greens, tops, or tips), as a fresh vegetable with rich nutrition and strong health promotion, can substantially improve the availability of foods (Islam, 2006). In 2006, the World Health Organization (WHO) voted sweetpotato as the champion of “the 10 best vegetables” (Ren et al., 2012), and it has also long been selected by the US National Aeronautics and Space Administration (NASA) as one of the primary food sources in controlled life-support systems (Hoff et al., 1982). Moreover, sweetpotato is used in traditional systems of medicine for preventing and treating various diseases, such as diabetes, hypertension, tumors, cancer, cardiovascular disease, inflammation, and aging (Behera et al., 2022). For the diversified applications, in addition to being a key functional food, sweetpotato is also mainly used as fodder, industrial raw materials, and energy source. Therefore, sweetpotato is indispensable for human nutrition and can help address the problems of food crisis, energy supply, and climate change.

Due to the biological importance of sweetpotato, its multi-dimensional research has attracted more and more attention from scholars, and the number of related articles has increased rapidly and greatly. Therefore, a summary analysis of the sweetpotato-related articles is necessary. Although many reviews on the research progress of sweetpotato have been published, they are mainly based on limited documents and summarized from different perspectives and directions in isolation. For example, the representative review topics include tuberous root development and starch (Lyu et al., 2021; Yang et al., 2023b), biotic and abiotic stress response (Yang et al., 2023a; Ahmed et al., 2024), anthocyanins, carotenoids, and the health-promoting functions (Li et al., 2019a; Alam, 2021; Tang et al., 2023), and genetics and genomics (Yan et al., 2022). However, these reviews cannot comprehensively and objectively reflect the whole picture of sweetpotato research; nor can they systematically exhibit the development process.

Bibliometrics can quickly draw and visualize the structures and dynamics of a research field by integrating numerous published articles, which can effectively reflect the current situation and future development of the subject. The comprehensive visual analysis software CiteSpace was found to be more suitable for statistical analysis than other commonly used software including HistCite, RefViz, SATI, and VOSviewer (Liu et al., 2022b). Herein, CiteSpace was employed to generate co-occurrence, co-citation, and timeline graphs to comprehensively visualize the structures and dynamics of sweetpotato research over the past 30 years (1993–2022), as only a little progress was made before 1993. The current review systemically summarizes the research progress and hotspots of sweetpotato, and identifies the key points and change trends through the econometric analysis, which will facilitate a comprehensive understanding of the development dynamics of sweetpotato research.

## Materials and methods

Web of Science (WoS) Core Collection database, as a globally recognized authoritative citation database, was searched for relevant articles published from January 1, 1993, to December 31, 2022. The search applied Boolean operator, Topic Search (including Title, Abstract, Author keywords, and Keywords Plus) = “sweet potato” OR sweetpotato OR “sweet potatoes” OR sweetpotatoes OR “*Ipomoea batatas*”. In total, 8,323 records, including 7,866 articles, 457 reviews, 63 highly cited articles, and one hot article, were exported with “Full Record and Cited References”. Then, the comprehensive retrospective analysis and summary were conducted using the CiteSpace software (Chen, 2006). Data processing adopted the theoretical framework of cluster analysis described in a previous report (Liu et al., 2022b). Briefly, the time partition was 1 year, the source included all items, Top = 50 for the threshold was selected, and others had default values. The keywords, authors, and references of the paper data units were selected as the node type. Critical path methods were applied to analyze the data collection element and draw the knowledge map, co-occurrence maps were employed to analyze the research hotspot, and time zone views were used to achieve development relationships.

## Results and discussion

### Number of publications and key research countries

**Figure 1:**
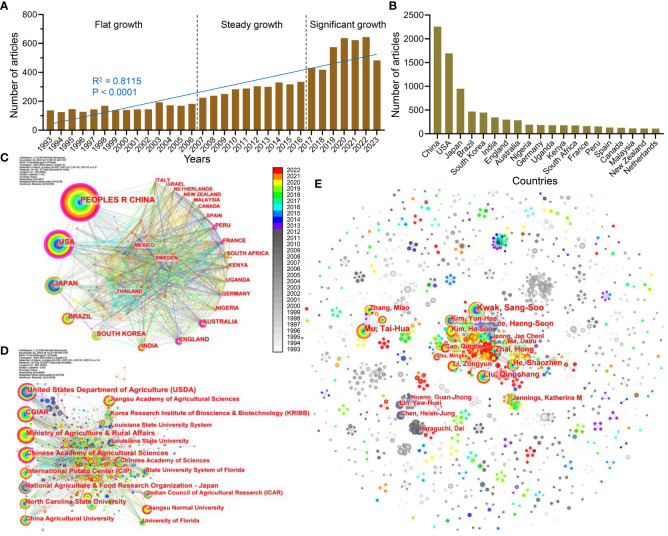
Publication trend and cooperation network analysis of sweetpotato-related articles. **(A)** The annual number of papers on sweetpotato research in the past 30 years. **(B)** Top 20 countries in the publication volume of sweetpotato research. **(C–E)** Network diagram of national cooperation **(C)**, institutional cooperation **(D)**, and author’s cooperation **(E)** in the sweetpotato research. The larger the node, the higher the amount of articles. The more connections between the two nodes, the stronger their cooperative connections. The rings of different colors represent different years as shown in the color chrominance card, and the ring thickness is proportional to the number of articles for a given time. The outermost purple ring of the node represents the centrality value calculated by the computed node centrality function of CiteSpace tool.

The annual number of publications can intuitively reveal the rate of development and change in specific fields. The statistics of sweetpotato-related articles reveal that the annual volume was relatively small (all less than 200 articles) and growing slowly from 1993 to 2006; thus, sweetpotato research has experienced a long period of flat growth. Subsequently, the annual number of publications accelerated steadily from 224 articles in 2007 to 334 articles in 2016. Comparatively, the publication numbers increased sharply from 2017 to 2022, and over 600 articles were published after 2020. As of November 1, 2023, 483 articles have been published (**Figure 1A**). The increasing numbers firmly exhibit that sweetpotato research is receiving increasing attention.

Then, through a comparative analysis of the publications, the key countries and institutions with large publications on sweetpotato research were identified. From 1993 to 2022, a total of 147 countries/regions and 724 institutions published articles about sweetpotato. The top 10 were China, the USA, Japan, Brazil, South Korea, India, the UK, Australia, Nigeria, and Germany, which account for approximately 65.4% of the total number of articles, and Asia accounts for 56.2% of the top 10, illustrating the important contribution of Asian countries/regions to the sweetpotato research (**Figure 1B**). China was the leading country (2,258 articles), accounting for 20.1% and 56.5% of the world and Asia, respectively. Although the USA ranks only eighth in sweetpotato production (Bach et al., 2021), the USA (1695 articles) and China occupy the dominant position in the sweetpotato research.

### Cooperation network analysis

Subsequently, the cooperative relationships between countries were analyzed using the CiteSpace software. The centrality degree is of great significance for the cooperative network analysis, and high centrality reflects that the node has a key influence on the relationship in the whole network. The results show that the USA had the highest centrality degree (0.32). Interestingly, although the co-occurrence frequencies of France and Australia were only 161 and 285 papers, respectively, their centrality degrees reached 0.18 and 0.17, respectively, even exceeding those of China (0.13). Furthermore, all countries with a high degree of centrality had a close cooperative relationship, and similar situations were observed in the subsequent analysis of institutional partnerships. Moreover, the early accumulation of sweetpotato research in the USA and Japan was more than that in China (before 2014) but was then quickly surpassed by China (**Figures 1C, D**).

The top 20 institutions in sweetpotato research are shown in **Supplementary Table S1**, of which China and the USA had the largest number of institutions, with eight and six, respectively. Among them, the United States Department of Agriculture (USDA) ranked first (395), followed by the Consultative Group on International Agricultural Research (CGIAR; 391), Ministry of Agriculture and Rural Affairs (MARA; 298) of China, Chinese Academy of Agricultural Sciences (CAAS; 262), and International Center of Potato (CIP; 244). The USDA had the highest centrality degree (0.26), followed by CAAS (0.13), CGIAR (0.12), National Agriculture & Food Research Organization (NARO; 0.12) of Japan, and Chinese Academy of Sciences (0.11). Moreover, institutions such as MARA, CIP, North Carolina State University, and China Agricultural University are commensurate in their top ranking of publications, and all of their cooperative connections with other institutions are also close (**Supplementary Table S1**, **Figures 1C, D**).

Through the analysis of the author’s published articles and cooperation networks, over 1,300 authors involved in sweetpotato research were found. The top 10 prolific authors were all Chinese and South Korean scholars, with six and four, respectively. Among the top 20 scholars, except for two American scholars, all of them were from Asia (12 from China, five from South Korea, and one from Japan), strengthening that Asian countries/regions pay more attention and research on sweetpotato (**Supplementary Table S2**). The top 10 scholars have published 654 articles, and Kwak Sang-Soo from the Korea Research Institute of Bioscience and Biotechnology (KRIBB) has published 121 articles, ranking first among all scholars. Then, their cooperative relationships were explored, and both centralized and decentralized distribution patterns were found (**Figure 1E**). Due to the large number of authors and their significant dispersion, the author’s cooperation network is like a starry sky. The main cooperation links between Chinese scholars (such as Mu Tai-hua, Liu Qingchang, He Shaozhen, Ma Daifu, and Li Zongyun), South Korean scholars (such as Kwak Sang-Soo, Lee Haeng-Soon, Kim Yun-Hee, and Kim Ho Soo), and Chinese and South Korean scholars were detected, and many minority groups of scholars also have a close cooperative connection.

### Dynamic evolution analysis of research topics and trends

#### Discipline evolution analysis

**Figure 2:**
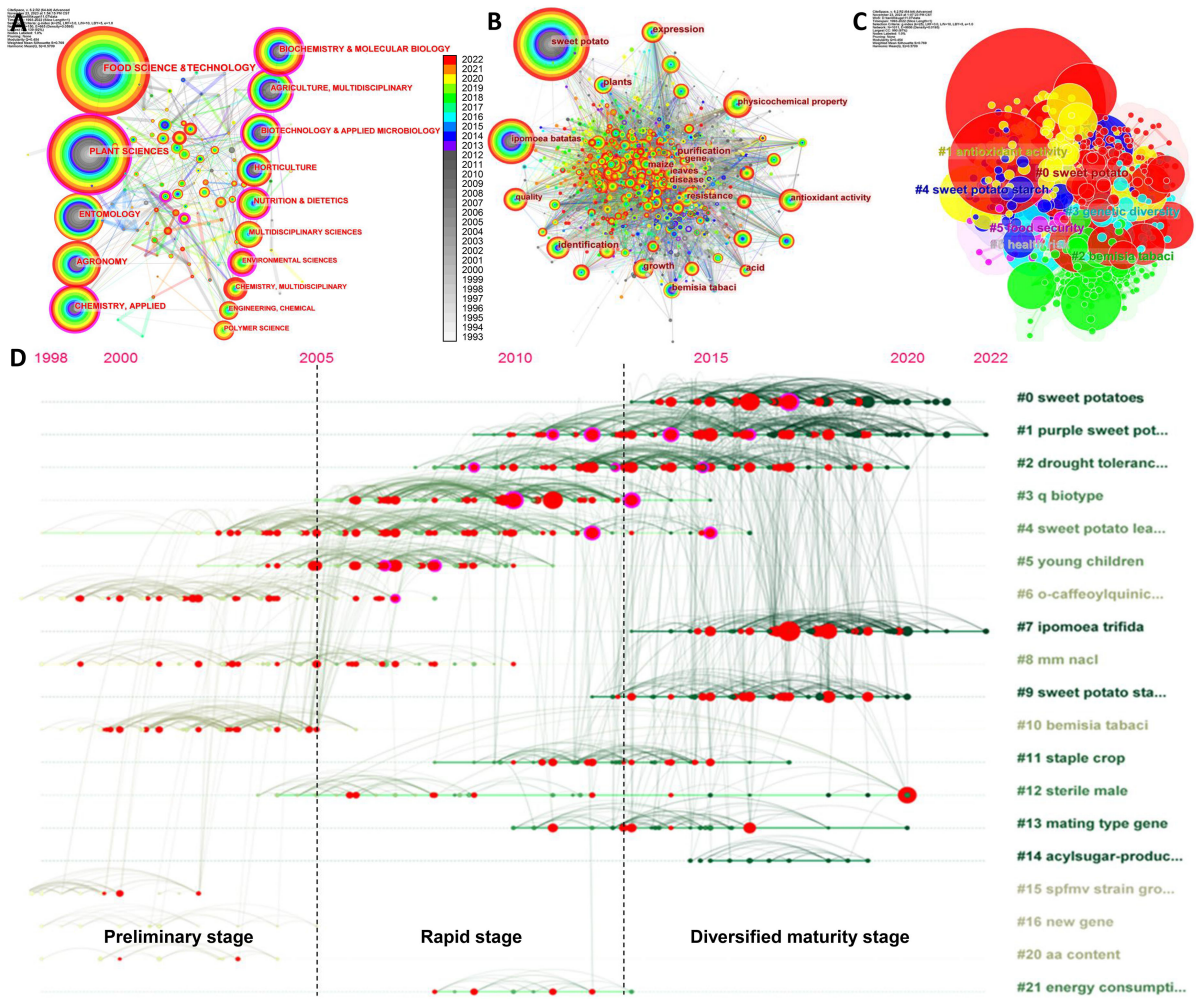
Analysis of disciplinary network, keyword co-occurrence, and timeline distribution of keywords. **(A)** Disciplinary network in the field of sweetpotato research from 1993 to 2022. **(B)** Keyword co-occurrence network in the field of sweetpotato research (1993–2022). The nodes represent the corresponding disciplines/keywords, and the node size is proportional to the number. The connection lines between nodes represent their relationships. The rings of different colors represent different years as shown in the color chrominance card, and the ring thickness is proportional to the number for a given time. The outermost purple ring of the node represents the centrality value calculated by the compute node centrality function of CiteSpace tool. **(C)** Co-occurrence clustering keyword network in the field of sweetpotato research. The serial number of clusters is inversely related to the number of their members; the smaller the number, the more members the cluster has. The keyword label with the largest value under the log-likelihood ratio (LLR) algorithm is used as the name of the cluster. **(D)** Timeline distribution of keywords for different topics. The vertical axis covers the same keywords of the same year, which shows the concerns of related fields in the same year. The horizontal axis concentrates the keywords of the same cluster, which displays the development result of the same cluster. The nodes and links in the graph display the inheritance and continuation of keywords, as well as the period between them.

The co-occurrence analysis of the subject network can establish the evolution of mainstream and interdisciplinary subjects of the research field. The results clearly show that sweetpotato research is a multidisciplinary field, in which Food Science & Technology and Plant Sciences are the main disciplines, which is closely related to the fact that sweetpotato is not only one of the main food crops worldwide but also used as an excellent model crop in basic research, followed by Entomology, Agronomy, Applied Chemistry, Biochemistry & Molecular Biology, and Agriculture, highlighting the multidisciplinary nature of sweetpotato research, which is inseparable from sweetpotato as an all-around key crop. Despite this, many of them do not display centralization characteristics, such as Food Science & Technology, Entomology, and Agronomy. On the contrary, Environmental Sciences (0.21), Biotechnology & Applied Microbiology (0.19), Analytical Chemistry (0.18), and Biochemistry & Molecular Biology (0.16) have the highest degree of centrality (**Figure 2A**, **Supplementary Table S3**).

Subsequently, the evolution of the top 20 discipline types emerging during 1993–2022 is listed in **Table 1**, which further revealed that sweetpotato research is a multidisciplinary cross. On average, there is a burst of discipline every 2 years, while the duration of different disciplines is different. Among them, Aerospace Engineering has the longest duration (15 years), followed by Cell Biology (11 years) and Biophysics (10 years), which are mainly concentrated in the early stages of sweetpotato research. Particularly, in the top 20 subject categories with the strongest citation bursts, there are multiple environment-related emerging disciplines, such as Environmental Sciences, Environmental Studies, and Environmental Engineering, suggesting that sweetpotato research is becoming more and more concerned in multiple disciplines.

**Table 1:** Top 20 discipline categories with the strongest citation bursts.

Discipline categories	Strength	Beginning	End	1993–2022
Entomology	61.93	1993	1997	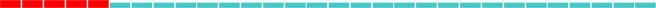
Plant Sciences	7.66	1993	1995	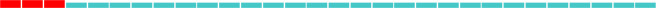
Horticulture	14.85	1993	1996	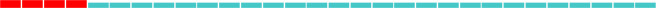
Agronomy	9.32	1998	1999	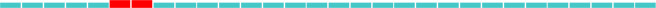
Zoology	6.32	1993	1999	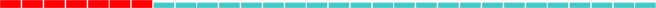
Chemistry, Analytical	6.68	1997	2002	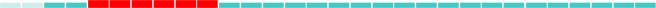
Cell Biology	15.96	1993	2003	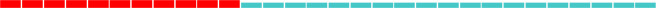
Biophysics	6.87	1996	2005	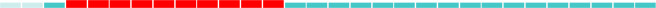
Biotechnology & Applied Microbiology	6.52	2008	2011	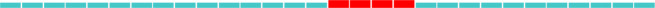
Agricultural Engineering	7.5	2008	2014	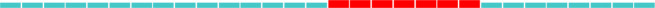
Virology	10.64	2008	2015	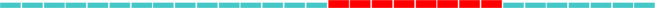
Multidisciplinary Sciences	21.79	2014	2018	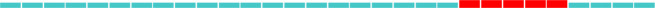
Chemistry, Multidisciplinary	23.13	2020	2022	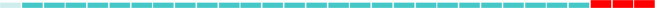
Polymer Science	14.57	2018	2022	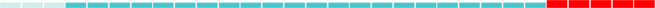
Environmental Sciences	10.7	2018	2022	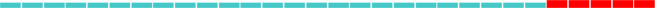
Physics, Applied	8.34	2019	2022	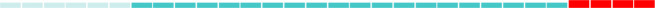
Materials Science, Multidisciplinary	7.39	2018	2022	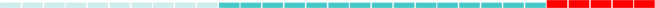
Environmental Studies	6.31	2018	2022	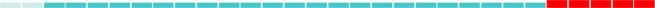
Engineering, Environmental	5.36	2020	2022	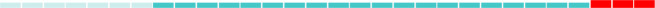
Green & Sustainable Science & Technology	5.07	2015	2022	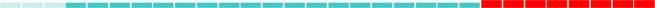

The time axis is indicated by blue lines, and the time interval of the outbreak is marked by red, which indicates the beginning year and the end year of the outbreak.

#### Co-occurrence network and timeline visualization analysis of keywords

Combined with the evolution of disciplines, the distribution of keywords was further analyzed. The co-occurrence network analysis of keywords can effectively reflect the development history and research hotspots. The intricate connection between approximately 1,000 keywords was found (**Figure 2B**), indicating the wide scopes and mature frameworks of sweetpotato research. In addition to the topic words sweetpotato and *I. batatas* (both have different spellings) having the largest nodes and complex network connections as expected, the top 10 keywords include Physicochemical property, Identification, Expression, *Bemisia tabaci*, Quality, Growth, Antioxidant activity, Plants, Resistance, and Cultivars, suggesting that they are the keywords with the most co-occurrences (**Supplementary Table S4**). Moreover, keyword clustering can reflect the main research clusters that have been formed in a certain field. The first six clusters represent the core of the sweetpotato research, including Sweetpotato, Antioxidant activity, *B. tabaci*, Antioxidant activity, Genetic diversity, Sweetpotato starch, Food security, and Health risk (**Figure 2C**), and the detailed keywords in each cluster are shown in **Supplementary Table S5**, illustrating their much broader relationships among the topics.

The refined keyword timeline diagram can reflect the evolution of research topics at different time stages. According to the node distribution and connecting lines, the diagram can be divided into three stages of development, which overlap with the stage division in publication trend analysis to a certain extent (**Figures 1A**, **2D**). The first stage (before 2005) has a small number of nodes with a small area that contains few themes and connecting lines, reflecting that the early research mainly focuses on several topics such as sweetpotato leaf, anthocyanin, salt stress, and *B. tabaci* and is in the preliminary stage. In the second stage of rapid development (2006–2013), both the number and area of nodes proliferated, the interconnection lines between nodes were dense, and multiple new keywords began to appear; the topics covered were still concentrated on a few keywords, and some continue to be inherited and some begin to weaken, such as purple sweetpotato, drought tolerance, whitefly, and sweetpotato leaf. In the third stage (2014–2022), sweetpotato research has shifted to both diversification and centralization. The number and area of nodes were further improved, the connections between nodes were denser, and most of the topics were covered. Similarly, some topics continue to be inherited, and some begin to weaken, such as the emerging concern of *Ipomoea trifida* and sweetpotato starch (**Figure 2D**). Therefore, the sweetpotato research in the present stage was wider in dimensions, deeper in scales, and more diverse in themes, which was in line with the results of co-occurrence analysis of disciplines and keywords, underscoring the increasing popularity of sweetpotato research.

#### Keyword burst analysis and research summary

Subsequently, CiteSpace was used to further detect citation burst keywords with high frequency and centrality as indicators of research frontiers. Approximately 330 such keywords were found, and the top 10 keywords for duration include Homoptera, Purification, *Bemisia argentifolii*, Hemiptera, Aleyrodidae, Sweetpotato whitefly, Cotton, Impact, *I. batatas*, and Cells (**Table 2**), suggesting their research hotspots. Among them, Aleyrodidae has the longest duration (23 years), followed by Homoptera, Amino acids, Populations, Coleoptera, Hymenoptera, and Lepidoptera (17–20 years), which are mainly concentrated in the early stages of sweetpotato research. The emerging keywords with the strongest citation bursts include Resistant starch, Impact, Rheological property, Abiotic stress, and digestibility. Based on the above analysis of research contents, the keyword timeline diagram was found to be more suitable for analyzing the hotspot evolution of sweetpotato research. Subsequently, the research topics of the published papers were manually screened, classified, and counted mainly by reading their abstract contents, and the representative papers closely related to sweetpotato biology research and cited in the forefront were focused on.

**Table 2:** Top 30 keywords with the most citation bursts.

Keywords	Strength	Beginning	End	1993–2022
Homoptera	26.41	1993	2012	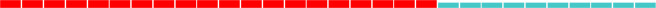
Aleyrodidae	18.79	1993	2015	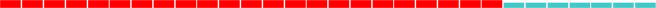
Sweet-potato whitefly	17.22	1993	1995	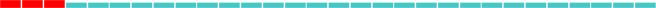
Cotton	16.85	1993	2008	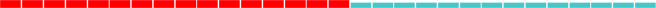
Cells	15.59	1993	2008	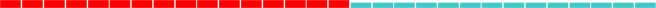
Populations	14.58	1993	2011	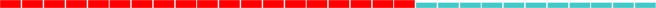
Curculionidae	14.45	1993	2006	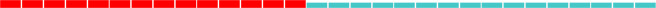
Sequence	11.42	1993	2000	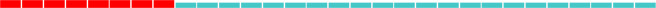
Genn	10.47	1993	2003	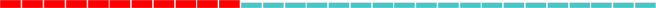
Invitro	10.27	1993	2002	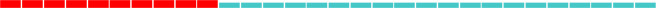
Cloning	10.22	1993	2001	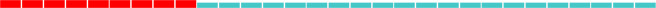
Coleoptera	11.02	1994	2012	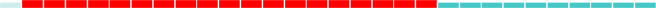
Nucleotide-sequence	10.11	1994	2008	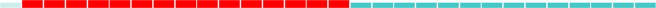
Purification	25.94	1995	2008	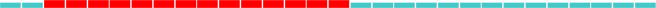
*Bemisia argentifolii*	22.83	1995	2002	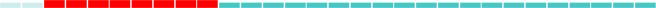
DNA	11.46	1996	2004	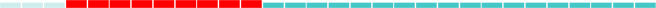
Polymerase chain reaction	10.24	1996	1999	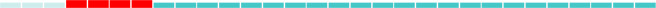
*Ipomoea batatas*	15.82	1998	2004	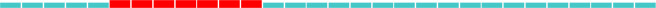
Amylopectin	10.11	2001	2007	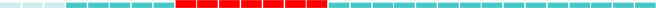
Hemiptera	19.97	2003	2014	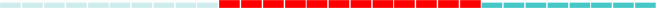
B biotype	15.42	2005	2015	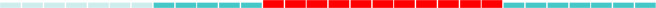
Insecticide resistance	12.85	2007	2013	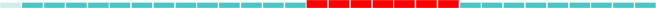
Beta-carotene	11.78	2013	2018	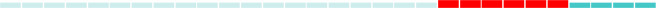
Purple sweetpotato	10.15	2015	2019	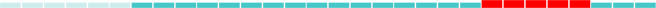
Resistant starch	9.95	2016	2022	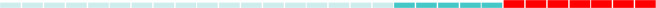
Impact	16.44	2017	2022	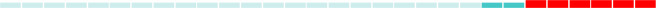
Rheological property	12.87	2018	2022	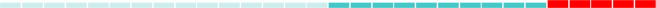
Abiotic stress	11.27	2018	2022	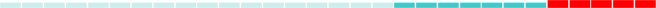
*In vitro* digestibility	10.81	2018	2022	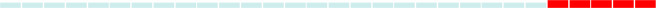
Digestibility	10.39	2018	2022	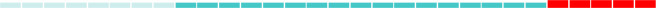

The time axis is indicated by blue lines, and the time interval of the keyword burst is marked by red, which indicates their beginning years and end years.

#### Preliminary stage (1993–2005)

During the preliminary accumulation stage, a total of 1,948 articles were published, including 51 review articles. During this period, the burst keywords related to whitefly were the highest, and the burst time was also the longest. Other keywords related to cells, DNA, sequence, virus, amylopectin, and caffeic acid were also found, indicating that related research had received significant attention in the early stage. For instance, biochemical, molecular, and whole-system approaches to study the potential diversity between reproductively isolated populations or biotypes of *B. tabaci* were systematically reviewed (Brown et al., 1995), and the migration, dispersal, and identification of sweetpotato whitefly *B. tabaci* have also been extensively explored (Byrne, 1999). Since sweetpotato whitefly is an effective vector for many viruses, sweetpotato virus disease (SPVD) and shoot tip culture of sweetpotato for virus elimination have also attracted corresponding attention (Gao et al., 2000; Tairo et al., 2005). Simultaneously, the beneficial effects of the antioxidant properties of sweetpotato chemical components on human health and nutrition have attracted much research (Cao et al., 1996; Philpott et al., 2004), and the efficacy of sweetpotato β-carotene has also made a small amount of progress (Van Jaarsveld et al., 2005). Sweetpotato starch has also received broad attention, but it mainly focuses on various physiological and biochemical characteristics (Zhang and Oates, 1999; Moorthy, 2002). Additionally, it is worth mentioning that the preface of sweetpotato genomics and molecular biology research has been opened at this stage, including the genetic diversity and relationship analysis of sweetpotato and its wild relatives, as well as their microsatellite sequence characterizations (Buteler et al., 1999; Huang and Sun, 2000), and several genes such as sweetpotato anionic POD gene *swpa1* and trypsin inhibitor gene *spTi-1* have been found to be associated with salt tolerance/oxidative stress tolerance and *Spodoptera litura* resistance, respectively (Yeh et al., 1997; Yun et al., 2000).

#### Rapid stage (2006–2013)

A total of 2,069 articles were published in the rapid stage, including 86 review articles and five highly cited articles were found. Compared with the preliminary accumulation stage, the continuous burst keywords related to whitefly, virus, β-carotene, flavonoids, and molecular cloning at this stage were found. Moreover, significant burst keywords such as insecticide resistance, ascorbate peroxidase, children, hydrogen peroxide, foods, rats/mice, mechanisms, and salt stress indicate the extensive depth of sweetpotato research. For example, host plants and natural enemies of the sweetpotato whitefly *B. tabaci* in China were investigated (Li et al., 2011), and the biotype-dependent secondary symbiont community in the sympatric population of sweetpotato whitefly *B. tabaci* and the global relationships of *B. tabaci* were studied (Boykin et al., 2007; Chiel et al., 2007). Moreover, the situation of sweetpotato whitefly in the UK and the prospects for developing eradication strategies were discussed (Cuthbertson et al., 2011). Correspondingly, research on sweetpotato viruses including their molecular detection was significantly improved (Kokkinos and Clark, 2006; Cuellar et al., 2009). For instance, the compositions of sweetpotato virus complexes, the effects of viruses on production, the biology of virus–plant interactions, and management approaches to viruses were summarized (Clark et al., 2012). Moreover, the comprehensive information on types of virus, yield loss mechanisms, increased yield, and benefit by cultivation of virus-free plants through meristem culture techniques and propagation system of virus-free seed tubers in China were systematically reviewed (Wang et al., 2010), and cryotherapy of shoot tips and the application in pathogen/virus eradication have also significantly served the preparation of healthy genetic resources (Wang and Valkonen, 2009; Feng et al., 2011).

Additionally, research on the benefits of sweetpotato to human health and nutrition, especially in antioxidant relevance, disease prevention, and treatment, has been further strengthened (Kurata et al., 2007; Teow et al., 2007; Padda and Picha, 2008). For instance, the beneficial effects on the health and nutrition of sweetpotato leaves were systematically reviewed and studied (Islam, 2006; Johnson and Pace, 2010). Ulteriorly, the characterization of anthocyanins and anthocyanidins in purple-fleshed sweetpotato was identified (Truong et al., 2010). In addition, more and more attention has been paid to research on carotenoids, such as their identification, content, and stability detection (Van Jaarsveld et al., 2006; Kimura et al., 2007). Moreover, changing the expression of carotenoid-related genes by genetic engineering not only can promote carotenoid accumulation but also can enhance the salt tolerance of transgenic sweetpotato (Kim et al., 2012, 2013). Therefore, this significantly promoted the application of orange-fleshed sweetpotato as a biofortified crop to prevent vitamin A deficiency.

Sweetpotato transgenic research has made great progress in the rapid stage, especially in the study of stress resistance. For instance, the overexpression of *CuZnSOD*, *IbLEA14*, *LOS5*, or *SPCP2* in sweetpotato/tobacco/*Arabidopsis* enhanced tolerance to various abiotic stresses, including salt, drought, and oxidative stress (Lee et al., 2007; Chen et al., 2010; Gao et al., 2011; Park et al., 2011). Moreover, molecular biology studies related to starch and tuberous root development have also made breakthroughs, and the functions of multiple related genes have been analyzed, including *IbEXP1*, *IbMADS1*, *SRD1*, *GBSSI*, and *SBEII* (Kitahara et al., 2007; Ku et al., 2008; Noh et al., 2010, 2013). With the advancement and popularization of omics technology, the era of multi-omics analysis of sweetpotato is also coming. During this period, various tissues/conditions underwent omics analysis including root transcriptomic/proteomic study (Lee et al., 2012; Firon et al., 2013), flower transcriptome (Tao et al., 2013), various tissues, and developmental stages (Tao et al., 2012), and transcriptomic and proteomic responses to whitefly (Yang et al., 2013).

#### Diversified maturity stage (2014–2022)

In the diversified maturity stage, a total of 4,306 articles (51.7% of the total) were published, including 320 review articles, 55 highly cited articles, and one hot article. There are many kinds of burst keywords such as Abiotic stress, Digestibility, Purple-fleshed sweetpotato, Resistant starch, Antioxidant, Health, Microstructure, Drought, Technology, Transcription factors, Carotenoids, Tuberous roots, Iron, Salt, Pigments, Amylose content, Biofortification, Overexpression, Reactive oxygen species, Model, and Molecular characterization, denoting that the biological research of sweetpotato is carried out comprehensively and in-depth at the current stage. Through reading and summarizing the research contents of published articles, it was found that studies on various aspects of sweetpotato have been involved in more and more in-depth mechanism explorations, which is inseparable from the divine assistance of its genomic information being decrypted. Due to the complexity of the genome of hexaploid sweetpotato, although the next-generation genome sequencing technology has been developed for more than a decade, its genome information has not made breakthrough progress until this period. Presently, the genome sequences of hexaploid cultivated species ‘Taizhong 6’ (a half haplotype-resolved hexaploid genome) (Yang et al., 2017) (https://sweetpotao.com/) and ‘Xushu 18’ (Yoon et al., 2022) (https://plantgarden.jp/ja/list/t4120/genome/t4120.G001), and two probable diploid wild relatives, *I. trifida* and *Ipomoea triloba* (http://sweetpotato.uga.edu/), are available (Hirakawa et al., 2015; Wu et al., 2018). Additionally, the complete chloroplast genomes of hexaploid and wild sweetpotato have also been sequenced (Yan et al., 2015; Wang et al., 2019b). This provides the most fundamental basis for the application of CRISPR/Cas technology, gene function analysis, molecular evolution resolution, and transcriptomic detection. Benefiting from this, research on the origin and diversity of sweetpotato from the molecular level is increasing in-depth, such as the genetic diversity, origin and evolution, allopolyploid discussion, and genome composition analysis (Muñoz-Rodríguez et al., 2018; Wadl et al., 2018). Of course, the current explorations based on specific locus amplified fragment sequencing (SLAF-seq), restriction-site associated DNA sequencing (RAD-seq), and simple sequence repeat (SSR) markers are also widely focused (Yang et al., 2015; Su et al., 2017; Feng et al., 2020). It is worth mentioning that the review article ‘Exploring and exploiting genetics and genomics for sweetpotato improvement: Status and perspectives’ is the present hot paper on sweetpotato research (Yan et al., 2022). Moreover, the acquisition of genomic information also provides the possibility for genome-wide gene family identification. Presently, approximately 35 different types of gene family papers have been published in hexaploid and its wild species, such as NAC (Guo et al., 2022), bZIP (Liu et al., 2022a), MADS-box (Shao et al., 2021) and SWEET (Dai et al., 2022), which lays a solid foundation for the standardized naming, evolutionary relationship, and functional analysis of genes.

During this period, the application of multi-omics in sweetpotato research, especially transcriptomic analysis, has made great progress. Approximately 85 relevant papers were published, mainly focusing on the response to abiotic stresses such as salt, drought, and low temperature (Ji et al., 2019; Zhu et al., 2019; Arisha et al., 2020; Meng et al., 2020), and anthocyanin biosynthesis and regulation (Qin et al., 2020; Li et al., 2021b; Zhang et al., 2022b), with approximately 25 and 15 papers, respectively. Moreover, many advances have also been made in biotic stress response (Lin et al., 2017; Lee et al., 2019), tuberous root formation and development (Wang et al., 2015; Ponniah et al., 2017; Dong et al., 2019), carotenoid biosynthesis (Li et al., 2015; Jia et al., 2022), and starch biosynthesis (Kou et al., 2020; Qin et al., 2021).

Moreover, many aspects of the rapid stage of research, such as whiteflies, viruses, flavonoids, and gene function identification, have made unprecedented significant progress. For example, the genome sequencing of the sweetpotato whitefly was completed (Xie et al., 2017), the insecticide resistance and management and control strategy of *B. tabaci* species were reviewed (Horowitz et al., 2020; Saurabh et al., 2021), and the invasion biology and management of sweetpotato whitefly in China were investigated (Guo et al., 2021). Correspondingly, research on SPVD and its prevention and control has also been continuously a hot topic, especially through molecular biology methods. Species and genetic variability of sweetpotato viruses in China (Liu et al., 2017; Wang et al., 2021) and virus incidence in Korea (Kim et al., 2017) were investigated, and the degeneration, decreased storage root yield and quality due to virus diseases, and their spread, transmission, and control were discussed (Gibson and Kreuze, 2015; Loebenstein, 2015; Hou et al., 2020). In detail, targeting SPCSV-*RNase3* by CRISPR-Cas13, inhibition of miR397 by short tandem target mimic (STTM) technology, and gene silencing induced by coat protein gene segments of various sweetpotato viruses could confer resistance against SPVD (Sivparsad and Gubba, 2014; Yu et al., 2022; Li et al., 2022a). In addition, new extensive progress has been made in the exploration and control of many other types of sweetpotato pests and bacterial pathogens, and multiple biotic stress resistance genes have been identified in this period, including *chit42* for white rot (Ojaghian et al., 2020), *IbBBX24* for Fusarium wilt (Zhang et al., 2020a), *IbMPK3/IbMPK6*-mediated *IbSPF1* for *Pseudomonas syringae* (Kim et al., 2019), *IbSWEET10* for *Fusarium oxysporum* (Li et al., 2017), and *IbNAC1* for pest *S. litura* (Chen et al., 2016). Furthermore, the exploration and control of sweetpotato weevil, parasitic nematodes, and root-knot nematodes have also achieved fruitful results (Kim and Yang, 2019; Okada et al., 2019; Nokihara et al., 2021).

More attention has been focused on research on the health-promoting functions of sweetpotato storage roots and leaves. The functionalities of various components such as carotenoids, anthocyanins, and caffeoylquinic acids were further determined through plenty of *in vitro* and *in vivo* assays. For example, the functional components of sweetpotato and their health effects, medicinal applications, and genetic improvements were comprehensively summarized in many review papers (Mohanraj and Sivasankar, 2014; De Albuquerque et al., 2019; Li et al., 2019a; Alam, 2021; Nguyen et al., 2021). Correspondingly, the chemical characterizations, nutritional functions, and antioxidant properties of roots and leaves were systematically studied (Sun et al., 2014b; Fu et al., 2016; Vishnu et al., 2019). In particular, the efficacy of anthocyanins in purple-fleshed sweetpotato in a series of diseases, including colon, bladder, and breast cancers (Li et al., 2018; Mazewski et al., 2018; Xu et al., 2018), inflammation (Sugata et al., 2015), hyperuricemia (Zhang et al., 2015), kidney and liver damage (Shan et al., 2014; Sun et al., 2014a), cognitive deficit (Zhuang et al., 2019), diabetes (Luo et al., 2021), and skin aging (Zhi et al., 2020) has attracted increasing attention and recognition from scholars. At the same time, many genes involved in their biosynthesis pathways and genetic modification/improvement have been identified, for instance, the IbMYB340-bHLH2-NAC56 complex (Wei et al., 2020), IbERF71 (Ning et al., 2021), and IbMYB44 (Li et al., 2021a) for anthocyanin biosynthesis and IbOr (Goo et al., 2015), IbLCYB2 (Kang et al., 2018), and IbGGPS (Li et al., 2022b) for carotenoid biosynthesis.

Furthermore, the development and popularization of sweetpotato transgenic technology have significantly improved the functional elucidation of related genes. The process of sweetpotato in response to environmental stresses has always been the focus and hotspot of research worldwide as described above. Among various abiotic stresses, salt and drought are the two main constraints for sweetpotato production, as most of them are produced in semi-arid regions (Solis Sarmiento, 2012). In this period, many successful stories about enhancing its drought and salt tolerance via genetic engineering have been reported. The encoding products of these genes are involved in a variety of pathways to confer sweetpotato resistance to salt and drought stress, mainly focusing on osmotic adjustments; for example, *IbPYL8*, *IbMIPS1*, and *IbPSS1* all confer sweetpotato salt and/or drought tolerance (Zhai et al., 2016; Yu et al., 2020; Xue et al., 2022); ion homeostasis, such as *IbNHX2*, could enhance salt and drought tolerance (Wang et al., 2016a); antioxidation, such as *CuZnSOD*, *APX*, *IbCAT2*, and *IbLCYB2*, improve stress tolerance through the enzymatic or non-enzymatic system (Yan et al., 2016; Yong et al., 2017; Kang et al., 2018); transcriptional regulation and signal transduction, such as *IbBBX24*, *IbbHLH66*, *IbNAC7*, *IbC3H18*, *IbSnRK1*, and *IbSIZ1a/b/c*, all positively regulate the stress tolerance (Zhang et al., 2019; Meng et al., 2020; Ren et al., 2020; Xue et al., 2022; Zhang et al., 2022a).

Furthermore, because sweetpotato is susceptible to chilling damage (4°C–10°C), the identification of cold stress tolerance genes began to receive particular attention at this stage, such as *IbMPK3* (Jin et al., 2022), *IbbHLH116* (Pan et al., 2022), and *IbCAD1* (Lee et al., 2021). Other stress resistance genes, such as those involved in the response to oxidative stress (Seo et al., 2015), heat stress (Ji et al., 2017), cadmium stress (Huo et al., 2018), and iron deficiency (Zhu et al., 2022), have also progressed. Moreover, functional explorations of genes related to tuberous root formation and development continue to show its hotspots, and the functions of multiple related genes have been revealed. For instance, the progress in molecular studies on storage root formation and physicochemical properties and molecular structures of starch in sweetpotato were summarized (Zhu and Wang, 2014; Tanaka, 2016), and genome-wide identification of candidate genes involved in storage root development was explored (Li et al., 2019b; Bararyenya et al., 2020). The roles of multiple genes in tuberous root development, such as *IbNAC083* (He et al., 2021), *IbRAP2.4* (Bian et al., 2022), *IbCAD1* (Lee et al., 2021), and *IbPAL1* (Yu et al., 2021), and starch biosynthesis, such as *IbSnRK1* (Ren et al., 2019), *IbVP1* (Fan et al., 2021), and *IbAATP* (Wang et al., 2016b), were assessed. Moreover, the knockout of starch biosynthesis genes *IbGBSSI* and *IbSBEII* via CRISPR/Cas9 to improve starch quality has been attempted (Wang et al., 2019a). This is the first application of CRISPR/Cas technology in sweetpotato breeding, which provides new opportunities for polyploid crops to accelerate breeding efficiency and increase yield and starch properties.

## Summary and prospects

As an economically important crop, sweetpotato has played a pivotal role in many aspects of its long history, such as food, energy, health, and environmental adaptation. In this review, the development history of sweetpotato research over the past 30 years is visually and comprehensively analyzed using the CiteSpace metrological analysis software, including the basic context, research priority, and development trend through the WoS database. Quantitative analysis and visual review show that Asia, especially China, has an important position in sweetpotato research. Although the USA has a small share of sweetpotato production, it is dominant in sweetpotato research and has a key influence in the global cooperation network. Contrarily, high sweetpotato yields in African countries do not match their research outputs. Generally, the research subjects of sweetpotato in China and abroad are roughly the same, but the contents and focus are different. Sweetpotato research in China involves more diverse research disciplines, covering a wide range of morphological, physiological, and biochemical responses and molecular dynamics studies, while the studies abroad pay more attention to its microscopic processes.

Keyword analysis shows primary research points and development directions in three main periods: preliminary stage (1993–2005), rapid stage (2006–2013), and diversified maturity stage (2014–2022). In recent years, sweetpotato research has developed rapidly, with unprecedented volume, diversity, and depth. Continuous development and innovation are in full swing in a multitude of fields of sweetpotato biology research, from genome sequencing to genetic improvement and application of genes related to tuberous root development, starch and pigment synthesis, and stress resistance; from *B. tabaci* and virus investigation to their prevention and control; and from anthocyanin and carotenoid identification to their antioxidant properties and health-promoting functions. Collectively, the general trend is gradually shifting from macro-scale to micro-scale and detailed exploration. Increasing studies have focused on revealing the relationships between morphological and physiological characteristics of sweetpotato and genetic information.

Although sweetpotato has incomparable advantages over other staple crops in solving the food crisis and climate change worldwide, its basic research is still seriously lagging compared with other staple crops such as rice, wheat, and maize. Through retrospective analysis, we can conclude that sweetpotato production still faces many thorny challenges and difficulties that require scholars’ continuous innovation and collaborative efforts to solve. 1) Accelerate the breeding efficiency of sweetpotato. The application of traditional breeding methods is obviously lagging behind due to the complicated polyploidy genome, limited flowering ability, and self- and cross-incompatibility of sweetpotato. As summarized above, in the past 15 years, with the continuous development of sweetpotato transgenic technology, sweetpotato genetic engineering has made remarkable progress, while more efficient and practical genetic transformation technology remains to be further developed, e.g., the cut-dip-budding delivery system (Cao et al., 2023). Furthermore, precise editing of starch biosynthesis genes via CRISPR/Cas9 and SPCSV-*RNase3* by CRISPR/Cas13 was shown to improve the starch quality and SPVD resistance in sweetpotato, respectively (Wang et al., 2019a; Yu et al., 2022), which will promote its breeding efficiency of genetic improvement. However, studies using the existing and subsequently improved CRISPR/Cas system to improve the related traits of sweetpotato are warranted. 2) Enhance the resistance to biotic and abiotic stresses. The yield and quality of sweetpotato are still observably affected by various adverse environmental conditions and virus diseases. The current study mainly focused on single stress or single genes, which will significantly limit the application of laboratory achievements in actual production. Therefore, more efforts should be made to research sweetpotato resistance to simultaneous abiotic stresses, crosstalk between abiotic and biotic stress signals, and co-expression of multiple genes. Additionally, the economical and applicable methods for large-scale production of disease-free sweetpotato materials through different types of tissue culture need to be continuously improved and explored. 3) Coordinate high yield with high quality and high resistance. Understanding the reciprocal regulation between stress-response and growth-control pathways is critical for resetting their balance and thus engineering stress-resistant and high-yield crops (Zhang et al., 2020b). With the significant development of agricultural production, although the yield should not be the primary goal of evaluating the quality of sweetpotato varieties (including edible quality, processing quality, and storage quality), no matter what type of sweetpotato, the yield traits must be considered. The current research isolated high yield, high quality, and high resistance, such as focusing only on the improvement of stress resistance while ignoring whether it affects yield or quality, or their opposite concerns. 4) Develop in-depth the health functions and added values. Promoting sweetpotato consumption is the original impetus for increasing its investment in multiple dimensions. Continuous and in-depth exploration of the health benefits and demands of sweetpotato is particularly critical, for instance, orange- and purple-fleshed sweetpotato in nutritional security and medicinal value; the development of new products including sweetpotato fries, wedges, bread, baked slices, and frozen products can enhance the industrial demand. Therefore, in order to cope with these challenges, long-term efforts and exhaustive research are needed under the synergistic mode of traditional and modern methods to cultivate elite sweetpotato varieties that reconcile high resistance and high quality with high yield.

## Author contributions

XM: Funding acquisition, Investigation, Software, Validation, Writing – original draft. TD: Funding acquisition, Writing – review & editing. ZL: Funding acquisition, Writing – review & editing. MZ: Conceptualization, Formal analysis, Funding acquisition, Investigation, Software, Supervision, Writing – original draft, Writing – review & editing.
