# Supplementary material for: First systematic review of the last 30 years of research on sweetpotato: elucidating the frontiers and hotspots
Source: Front Plant Sci. 2024 Jul 4;15:1428975. doi: 10.3389/fpls.2024.1428975 (PMC11258629; doi:10.3389/fpls.2024.1428975)
Supplement: Supplementary file 1 [file Table_1.docx]

**Supplementary Table S1**. Top 20 institutions in sweetpotato-related publications from 1993 to 2022.

| Number | Affiliations | Counts | Centrality |
| --- | --- | --- | --- |
| 1 | United States Department of Agriculture (USDA) | 395 | 0.26 |
| 2 | Consultative Group on International Agricultural Research (CGIAR) | 319 | 0.12 |
| 3 | Ministry of Agriculture Rural Affairs (MARA) | 298 | 0.06 |
| 4 | Chinese Academy of Agricultural Sciences (CAAS) | 262 | 0.13 |
| 5 | International Potato Center (CIP) | 244 | 0.06 |
| 6 | National Agriculture & Food Research Organization (NARO) | 230 | 0.12 |
| 7 | North Carolina State University | 209 | 0.03 |
| 8 | China Agricultural University | 159 | 0.06 |
| 9 | Jiangsu Academy of Agricultural Sciences | 156 | 0.05 |
| 10 | Korea Research Institute of Bioscience & Biotechnology (KRIBB) | 150 | 0.04 |
| 11 | Louisiana State University System | 146 | 0.02 |
| 12 | Louisiana State University | 137 | 0.02 |
| 13 | Chinese Academy of Sciences | 133 | 0.11 |
| 14 | State University System of Florida | 125 | 0.04 |
| 15 | Indian Council of Agricultural Research (ICAR) | 120 | 0.02 |
| 16 | Jiangsu Normal University | 114 | 0.02 |
| 17 | University of Florida | 106 | 0.01 |
| 18 | Academia Sinica | 98 | 0.01 |
| 19 | University of Greenwich | 92 | 0.03 |
| 20 | National Taiwan University | 89 | 0.04 |

**Supplementary Table S2**. Top 60 authors in the cooperation network analysis of sweetpotato-related publications from 1993 to 2022.

| Number | Authors | Sizes | Affiliations | Countries |
| --- | --- | --- | --- | --- |
| 1 | Kwak, Sang-Soo | 121 | Korea Research Institute of Bioscience & Biotechnology (KRIBB) | South Korea |
| 2 | Mu, Tai-Hua | 89 | Chinese Academy of Agricultural Sciences | China |
| 3 | Liu, Qingchang | 70 | China Agricultural University | China |
| 4 | Lee, Haeng-Soon | 63 | Korea Research Institute of Bioscience & Biotechnology (KRIBB) | South Korea |
| 5 | Zhai, Hong | 62 | China Agricultural University | China |
| 6 | He, Shaozhen | 62 | China Agricultural University | China |
| 7 | Lin, Yaw-Huei | 54 | Academia Sinica | China |
| 8 | Kim, Yun-Hee | 47 | Gyeongsang National University | South Korea |
| 9 | Li, Zongyun | 43 | Jiangsu Normal University | China |
| 10 | Kim, Ho Soo | 43 | Korea Research Institute of Bioscience & Biotechnology (KRIBB) | South Korea |
| 11 | Zhang, Miao | 41 | Chinese Academy of Agricultural Sciences | China |
| 12 | Jennings, Katherine M | 35 | North Carolina State University | USA |
| 13 | Ma, Daifu | 34 | Chinese Academy of Agricultural Sciences | China |
| 14 | Haraguchi, Dai | 32 | Okinawa Prefectural Plant Protection Center | Japan |
| 15 | Chen, Hsien-Jung | 31 | National Sun Yat-sen University | China |
| 16 | Huang, Guan-Jhong | 30 | China Medical University | China |
| 17 | Cao, Qinghe | 30 | Chinese Academy of Agricultural Sciences | China |
| 18 | Jeong, Jae Cheol | 30 | Korea Research Institute of Bioscience & Biotechnology (KRIBB) | South Korea |
| 19 | Monks, David W | 29 | North Carolina State University | USA |
| 20 | Sun, Jian | 29 | Jiangsu Normal University | China |
| 21 | Kumano, Norikuni | 28 | Obihiro University of Agriculture and Veterinary Medicine | Japan |
| 22 | Zhang, Min | 28 | Jiangnan University | China |
| 23 | Clark, Christopher A | 28 | Louisiana State University Agricultural Center | USA |
| 24 | Brown, Judith K | 27 | University of Arizona | USA |
| 25 | Yamakawa, O | 26 | National Agricultural Research Center for Kyushu Okinawa Region | Japan |
| 26 | Nakamura, K | 26 | Niigata University Graduate School of Medical and Dental Sciences | Japan |
| 27 | Noda, Takahiro | 26 | Hokkaido Agricultural Research Center | Japan |
| 28 | Sun, Hongnan | 25 | Chinese Academy of Agricultural Sciences | China |
| 29 | Hou, Wen-Chi | 25 | Taipei Medical University | China |
| 30 | Kuriwada, Takashi | 23 | Kagoshima University | Japan |
| 31 | Kohama, Tsuguo | 22 | Okinawa Prefectural Plant Protection Center | Japan |
| 32 | Ji, Chang Yoon | 22 | Korea Research Institute of Bioscience & Biotechnology (KRIBB) | South Korea |
| 33 | Zhao, Ning | 22 | China Agricultural University | China |
| 34 | Tang, Zhonghou | 21 | Chinese Academy of Agricultural Sciences | China |
| 35 | Park, Sung-Chul | 21 | Korea Research Institute of Bioscience & Biotechnology (KRIBB) | South Korea |
| 36 | Kou, Meng | 21 | Chinese Academy of Agricultural Sciences | China |
| 37 | Mwanga, Robert O M | 21 | International Potato Centre | Uganda |
| 38 | Xu, Tao | 21 | Jiangsu Normal University | China |
| 39 | Gibson, Richard W | 21 | International Potato Centre (CIP) | Uganda |
| 40 | Yoshimoto, Makoto | 21 | National Agricultural Research Center for Kyusyu Okinawa Region | Japan |
| 41 | Bian, Xiaofeng | 20 | Jiangsu Academy of Agricultural Sciences | China |
| 42 | Terahara, Norihiko | 19 | Minami-Kyushu University | Japan |
| 43 | Simmons, Alvin M | 18 | United States Department of Agriculture (USDA) | USA |
| 44 | Zhang, Youjun | 18 | Chinese Academy of Agricultural Sciences | China |
| 45 | Yencho, G Craig | 18 | North Carolina State University | USA |
| 46 | Dong, Tingting | 18 | Jiangsu Normal University | China |
| 47 | Li, Qiang | 18 | Chinese Academy of Agricultural Sciences | China |
| 48 | Zhang, Peng | 17 | Chinese Academy of Sciences | China |
| 49 | Kim, Sun-Hyung | 17 | University of Seoul | South Korea |
| 50 | Schultheis, Jonathan R | 17 | North Carolina State University | USA |
| 51 | Low, Jan W | 17 | International Potato Centre (CIP) | Kenya |
| 52 | Meyers, Stephen L | 16 | Mississippi State University | USA |
| 53 | Zhu, Mingku | 16 | Jiangsu Normal University | China |
| 54 | Ahn, Mi-Jeong | 16 | Gyeongsang National University | South Korea |
| 55 | Liu, Jun | 16 | Yangzhou University | China |
| 56 | Kwon, Suk-Yoon | 15 | Korea Research Institute of Bioscience & Biotechnology (KRIBB) | South Korea |
| 57 | Zhang, Huan | 15 | China Agricultural University | China |
| 58 | Shiromoto, Keiko | 15 | Okinawa Prefectural Plant Protection Center | Japan |
| 59 | Chaudhari, Sushila | 15 | North Carolina State University/Michigan State University | USA |
| 60 | Xie, Wen | 15 | Chinese Academy of Agricultural Sciences | China |

**Supplementary Table S3**. Top 30 subjects in the co-occurrence network analysis of sweetpotato-related research from 1993 to 2022.

| Number | Subject name | Counts | Centrality |
| --- | --- | --- | --- |
| 1 | Food Science & Technology | 2058 | 0.09 |
| 2 | Plant Sciences | 1548 | 0.13 |
| 3 | Entomology | 863 | 0.01 |
| 4 | Agronomy | 817 | 0.05 |
| 5 | Chemistry, Applied | 794 | 0.1 |
| 6 | Biochemistry & Molecular Biology | 707 | 0.16 |
| 7 | Agriculture, Multidisciplinary | 607 | 0.14 |
| 8 | Biotechnology & Applied Microbiology | 540 | 0.19 |
| 9 | Horticulture | 480 | 0 |
| 10 | Nutrition & Dietetics | 458 | 0.12 |
| 11 | Multidisciplinary Sciences | 258 | 0.01 |
| 12 | Environmental Sciences | 223 | 0.21 |
| 13 | Chemistry, Multidisciplinary | 192 | 0.1 |
| 14 | Engineering, Chemical | 191 | 0.04 |
| 15 | Polymer Science | 188 | 0.05 |
| 16 | Genetics & Heredity | 186 | 0.02 |
| 17 | Virology | 126 | 0.01 |
| 18 | Soil Science | 115 | 0.01 |
| 19 | Microbiology | 113 | 0.03 |
| 20 | Zoology | 107 | 0.06 |
| 21 | Agriculture, Dairy & Animal Science | 107 | 0.01 |
| 22 | Biology | 99 | 0.07 |
| 23 | Ecology | 98 | 0.08 |
| 24 | Chemistry, Analytical | 97 | 0.18 |
| 25 | Pharmacology & Pharmacy | 96 | 0.09 |
| 26 | Cell Biology | 93 | 0.08 |
| 27 | Chemistry, Medicinal | 90 | 0.01 |
| 28 | Agricultural Engineering | 87 | 0 |
| 29 | Chemistry, Organic | 80 | 0 |
| 30 | Energy & Fuels | 79 | 0.11 |

**Supplementary Table S4**. Top 60 keywords in the co-occurrence network analysis of sweetpotato-related research from 1993 to 2022.

| Number | Keyword name | Counts | Centrality |
| --- | --- | --- | --- |
| 1 | Sweet potato | 2432 | 0.08 |
| 2 | *Ipomoea batatas* | 1068 | 0.03 |
| 3 | Physicochemical property | 433 | 0.02 |
| 4 | Identification | 384 | 0.09 |
| 5 | Expression | 378 | 0.05 |
| 6 | *Bemisia tabaci* | 339 | 0.05 |
| 7 | Quality | 334 | 0.02 |
| 8 | Growth | 331 | 0.05 |
| 9 | Antioxidant activity | 313 | 0.02 |
| 10 | Plants | 285 | 0.05 |
| 11 | Resistance | 271 | 0.05 |
| 12 | Cultivars | 222 | 0.03 |
| 13 | Oxidative stress | 221 | 0.02 |
| 14 | Protein | 221 | 0.04 |
| 15 | Starch | 211 | 0.02 |
| 16 | Functional property | 190 | 0.02 |
| 17 | Purple sweet potato | 185 | 0.01 |
| 18 | Yield | 178 | 0.01 |
| 19 | Rice | 178 | 0.03 |
| 20 | Acid | 171 | 0.05 |
| 21 | Aleyrodidae | 163 | 0.03 |
| 22 | Anthocyanins | 162 | 0.01 |
| 23 | Gene | 159 | 0.05 |
| 24 | Homoptera | 159 | 0.03 |
| 25 | Arabidopsis | 159 | 0.02 |
| 26 | Temperature | 158 | 0.03 |
| 27 | Purification | 157 | 0.07 |
| 28 | Food | 152 | 0.02 |
| 29 | Leaves | 144 | 0.05 |
| 30 | Beta carotene | 143 | 0.02 |
| 31 | Storage | 139 | 0.01 |
| 32 | Metabolism | 139 | 0.03 |
| 33 | Sweet potato starch | 137 | 0.01 |
| 34 | Gelatinization | 137 | 0.01 |
| 35 | Diversity | 136 | 0.02 |
| 36 | Biosynthesis | 136 | 0.02 |
| 37 | Maize | 124 | 0.05 |
| 38 | *Arabidopsis-thaliana* | 124 | 0.02 |
| 39 | Biological control | 123 | 0.02 |
| 40 | Stability | 122 | 0.01 |
| 41 | Gene expression | 120 | 0.03 |
| 42 | Amylose | 120 | 0.01 |
| 43 | Disease | 118 | 0.06 |
| 44 | Wheat | 114 | 0.03 |
| 45 | Genetic diversity | 114 | 0.01 |
| 46 | Evolution | 112 | 0.03 |
| 47 | Populations | 111 | 0.04 |
| 48 | Accumulation | 110 | 0.02 |
| 49 | Digestibility | 108 | 0.01 |
| 50 | Antioxidant | 108 | 0.01 |
| 51 | Management | 108 | 0.03 |
| 52 | Rheological property | 100 | 0.01 |
| 53 | Impact | 100 | 0.01 |
| 54 | Roots | 98 | 0.03 |
| 55 | Resistant starch | 96 | 0 |
| 56 | Color | 91 | 0.02 |
| 57 | Responses | 91 | 0.01 |
| 58 | Tolerance | 90 | 0.01 |
| 59 | Cells | 87 | 0.03 |
| 60 | Kinetics | 87 | 0.01 |

**Supplementary Table S5**. The first six keyword clusters in sweetpotato-related research from 1993 to 2022. The silhouette value greater than 0.6 was considered statistically significant. The keyword label with the largest value under the LLR algorithm is used as the name of the cluster.

| Cluster ID | Size | Silhouette value | Average Year | Label (LLR algorithm) |
| --- | --- | --- | --- | --- |
| #0 | 251 | 0.781 | 2002 | sweet potato (189.35, 1.0E-4);  gene expression (126.43, 1.0E-4);  *Ipomoea batatas* (125.06, 1.0E-4);  sweetpotato (117.52, 1.0E-4);  *Bemisia tabaci* (107.73, 1.0E-4) |
| #1 | 234 | 0.669 | 2011 | antioxidant activity (167.33, 1.0E-4);  purple sweet potato (158.87, 1.0E-4);  anthocyanins (134.95, 1.0E-4);  *Bemisia tabaci* (87.77, 1.0E-4);  polyphenols (60.5, 1.0E-4) |
| #2 | 192 | 0.833 | 2001 | *Bemisia tabaci* (452.62, 1.0E-4);  sweetpotato whitefly (145.4, 1.0E-4);  *Bemisia argentifolii* (122.33, 1.0E-4);  biological control (122.33, 1.0E-4);  aleyrodidae (106.47, 1.0E-4) |
| #3 | 145 | 0.737 | 2004 | genetic diversity (104.22, 1.0E-4);  feathery mottle virus (76.04, 1.0E-4);  crinivirus (68.01, 1.0E-4);  SPFMV (59.99, 1.0E-4);  genome (51.98, 1.0E-4) |
| #4 | 130 | 0.836 | 2010 | sweet potato starch (255.37, 1.0E-4);  starch (205.73, 1.0E-4);  physicochemical properties (142.88, 1.0E-4);  pasting properties (102.26, 1.0E-4);  resistant starch (102.26, 1.0E-4) |
| #5 | 33 | 0.857 | 2012 | food security (39.57, 1.0E-4);  soil properties (38.22, 1.0E-4);  climate change (31.84, 1.0E-4);  poultry manure (25.46, 1.0E-4);  biochar (25.46, 1.0E-4) |
| #6 | 5 | 0.981 | 2021 | health risk (33.26, 1.0E-4);  heavy metals (33.26, 1.0E-4);  food crops (22.12, 1.0E-4);  cadmium (13.78, 0.001);  vegetable (12.17, 0.001) |
